# Oral and general health conditions involved in periodontal status during pregnancy: a prospective cohort study

**DOI:** 10.1007/s00404-022-06843-3

**Published:** 2022-12-13

**Authors:** J. A. Gil-Montoya, T. Rivero-Blanco, X. Leon-Rios, M. Exposito-Ruiz, I. Pérez-Castillo, M. J. Aguilar-Cordero

**Affiliations:** 1 Granada School of Dentistry, Institute of Biomedical Research of Granada, University of Granada, c/ Paseo de Cartuja S/N, 18071 Granada, Spain; 2 Granada School of Dentistry, University of Granada, Granada, Spain; 3 School of Dentistry, Universidad Peruana de Ciencias Aplicadas, Santiago de Surco, Perú; 4 Faculty of Health Sciences, University of Granada, Granada, Spain; 5 Faculty of Health Sciences, Department of Nursing, University of Granada, Granada, Spain; 6 Andalusian Plan for Research Development and Innovation, University of Granada, CTS 367, Granada, Spain

**Keywords:** Oral health, Pregnancy-related periodontal status, Pregnancy, Obesity, Oral hygiene

## Abstract

**Purpose:**

Pregnancy is a period in a woman’s life that has important consequences on oral health, particularly for gingival health. Present study aims to identify women at higher risk of developing periodontal disease (gingivitis and periodontitis) during late pregnancy and evaluate how this condition evolves during this period.

**Methods:**

Prospective cohort study was designed with pregnant women who were assessed during the first and third trimesters of gestation in a southern Spanish public hospital. Data regarding gingival and periodontal health, oral hygiene, and overall health status (obesity and diabetes mellitus) were collected. Reporting followed STROBE checklist.

**Results:**

Significantly higher number of women had the periodontal and gingival disease in the third trimester of gestation compared with in early pregnancy. In the third trimester of gestation, 42 (28.6%) and 63 (42.9%) of women presented symptoms of periodontal disease and gingival disease, respectively. Obesity (OR 2.834; 95%CI 0.919–8.741), worse oral hygiene during the first trimester of gestation (OR: 4.031; 95%CI 2.12–7.65), and periodontal disease during early pregnancy (OR: 15.104; 95%CI 3.60–63.36) most effectively predicted periodontal disease during late pregnancy.

**Conclusions:**

Pregnancy is associated with exacerbated periodontal and gingival disease symptoms throughout the different trimesters of gestation. Obesity and oral hygiene during early pregnancy were the risk factors that most contributed to the aforementioned changes in periodontal disease.

## What does this study add to the clinical work

**Table:** 

Obesity and oral hygiene during early pregnancy seems to be risk factors that most contributed to the association between exacerbated periodontal disease and gestation.	

## Introduction

Women undergo physiological changes during pregnancy, which have an impact on the structure of the oral cavity due to increased levels of estrogen and progesterone [1]. Gingivitis and periodontitis are the most prevalent oral disorders [2], but other conditions can arise, namely, dental caries, tooth erosion, xerostomia (subjective dry mouth symptoms), and benign tumors such as epulis fissuratum [3]. There is a wide consensus that oral health should be an integral part of preventive health care for pregnant women and their newborns [4]. It is important to advise about the various changes that occur to the gums and teeth during pregnancy and reinforce good oral health habits to keep the gingiva and teeth healthy, particularly in higher-risk pregnancies [5].

Periodontal disease (PD) is a common chronic inflammatory condition that is infectious. When the inflammation is confined to the soft tissue, this condition is called gingivitis, however, when the affected area extends to the connective tissue and the bone surrounding the tooth, the condition is referred to as periodontitis [6]. Gingival inflammation during pregnancy usually originates between the third and eighth months of gestation and abates during the last month of pregnancy, often disappearing after delivery [3]. It is plaque-induced [2] and characterized by gingival erythema, edema, hyperplasia, and increased bleeding [3]. Although the mechanisms responsible for increased gingival inflammation in pregnant women are not fully understood, perturbations in neutrophil function, alterations in cellular and humoral immunity, hormone-induced changes in cellular physiology, and local effects on microbial ecology all play important roles in the overall process [7]. On the other hand, periodontitis is a chronic multifactorial inflammatory disease associated with dysbiotic plaque biofilms and characterized by progressive destruction of the tooth-supporting tissues [8]. So far, it is unclear whether a pregnancy is related to a higher risk of periodontitis, which is a more severe state of periodontal disease, or only related to gingivitis or other clinical periodontal parameters [9]. During this period, other systemic conditions such as obesity [10, 11] and diabetes mellitus [12, 13], are also associated with alterations in periodontal tissues, as well as pregnancy itself.

According to scientific literature, bacteria plaque control during pregnancy and more complex periodontal treatments such as scaling and root planning, can contribute to decreased gingival inflammation [14], improved quality of life during pregnancy [15], and reduce the prevalence of adverse pregnancy outcomes in women with PD [16]. Also, early interventions in healthy and high-risk pregnant women can play a key role in the preservation of adequate periodontal health throughout pregnancy [17]. For these reasons, identifying groups of women at high risk of developing gingival and periodontal conditions during pregnancy (women with obesity and/or diabetes mellitus or women with periodontal diseases before pregnancy), is necessary to improve the efficiency of these dental interventions and directing the attention of the caregivers and health professionals responsible for these patients towards the implementation of early preventive measures. In light of the scientific literature, the present study aims to identify women at higher risk of developing PD (gingivitis and periodontitis) during late pregnancy and evaluate how PD evolves during this period.

## Methods

The present prospective cohort study was nested within a wider follow-up research program on pregnant women, although oral examinations and measurements were entirely independent with regard to both design and execution. The study was approved by the “Granada Local Ethics Committee, Spain”, reference number 72-2015/27-07-2018, and conducted in accordance with the principles of the Declaration of Helsinki. Written informed consent was obtained from all the study participants. The manuscript is reported following the STROBE checklist.

### Sample and setting

Women were recruited through the obstetrics and gynecology service of the University Hospital Virgen de las Nieves, Granada, in Spain. Specifically, this medical center dealt with 2956 deliveries in 2019. For the present research, pregnant women were recruited during 2018–2019 and follow-up between 10–12 and 30–32 weeks pregnant. Women were approached during their first routine prenatal check-ups and invited to participate in the study. Those showing interest were provided with further information. The sample size calculated for the initial study was 147 women. For the aim of this study (to create two multivariate models that would allow us to predict the presence of periodontitis and gingivitis in the third trimester of pregnancy), considering a minimum of 10 cases per independent variable is generally recommended, with a sample size of 147 women we could include up to 4 predictive variables in the periodontitis model (*P* = 0.28) and 6 in the gingivitis model (*P* = 0.43). These variables were calculated using the Peduzzi formula[18] [*n* = 10*k/P], where K is the number of independent variables to be calculated, *P* is the smallest of the proportions of negative or positive cases in the population and n is the number of relevant cases.

Pregnant women were selected in line with the following inclusion criteria: (1) aged older than 16 years, (2) at 10–12 weeks pregnant as assessed by ultrasonography, and (3) provide signed informed consent. Women with physical disabilities that could compromise study participation were excluded, as were those carrying multiple fetuses. Women with less than six teeth or who had been treated for periodontal disease in the six months prior to the research being undertaken were also excluded from study participation. Women with previous diagnoses of gingivitis or periodontitis were included in the study to perform a realistic analysis in a routine clinical setting. Of the 230 women who attended consultation during the study period, 24 declined participation due to a lack of interest. Of the 206 women who finally signed informed consent, 147 participants completed the follow-up period and were therefore considered for analysis. Reasons for study drop-out were that women had incomplete clinical records (*n* = 29), had received periodontal treatment during the first or third trimesters of gestation (*n* = 9), had been referred to the fetal assessment unit (*n* = 6), or did not attend the second evaluation during the third trimester of gestation for undisclosed reasons (*n* = 15).

### Oral assessment

Two experienced dentists previously trained in the evaluation of periodontal disease collected all data related to oral health throughout the study (80% inter-rater reliability for the assessment of clinical attachment loss and plaque index). Oral examinations were conducted at obstetrics and gynecology services under artificial light and using disposable mirrors, University of North Carolina periodontal probes and in line with WHO guidelines [19]. Data were collected regarding oral hygiene habits, namely tooth brushing (frequency of daily brushing), mouthwash use (regular or not regular), flossing (never, regularly, or only during pregnancy), and the DMFT index (sum of the number of decayed, missing and filling teeth). Bleeding on probing (BOP) was used to evaluate non-plaque-dependent active gingivitis (each tooth is probed and then scored as 0 or 1 depending on the absence or presence of bleeding) and the Silness and Löe plaque index [20] was used to assess oral hygiene. With regards to this last index, possible final scores range between 0 and 3 and are calculated by summing the scores obtained for each tooth and then dividing this outcome by the number of examined teeth. Finally, to evaluate the presence of periodontitis since their first visit, periodontal attachment loss and pocket depth were assessed for each tooth apart from the third molar. Cases of periodontitis were defined in accordance with Tonetti MS et al. [21]. Hence, periodontitis was diagnosed as a clinical attachment loss (CAL) (vestibular-palatine) of ≥ 3 mm and a pocket depth of > 3 mm in two or more teeth. Accordingly, gingivitis was defined as gingival inflammation along with bleeding on probing in 10% of the explored sites in the absence of pockets > 3 mm [21].

### General assessment

Participant sociodemographic data were obtained from questionnaires. Data included the following variables: age, educational level (elementary school, high school/vocational training, higher education or above), employment status (employed/unemployed), smoking (regular smoker-at least one cigarette in the last month-/never smokes), history of previous pregnancy outcomes, medical history, and drug prescription. During 24 weeks of pregnancy, all participants underwent O’Sullivan’s test to diagnose pregnancy hyperglycemia. Cases of pregnancy hyperglycemia were further examined using the glucose challenge test to diagnose cases of gestational diabetes mellitus.

Body mass index (BMI) was calculated to identify cases of obesity and overweight. This was done by dividing weight in kilograms by height squared in meters. The present study followed guidelines based on World Health Organization criteria. In this way, participants were categorized according to three groups: normal weight (BMI ≥ 18.5 to ≤ 24.9 kg/m^2^), overweight (BMI ≥ 25 to ≤ 29.9 kg/m^2^), and obese (BMI ≥ 30 kg/m^2^). Weight and height data were collected from medical records at the beginning of the study.

### Follow-up of participants

All participants underwent an oral evaluation during the first hospital appointment. Information regarding oral hygiene habits and possible pregnancy-related oral health conditions was provided to participants. The public healthcare system in Spain provides pregnant women with a close follow-up which consists of three routine prenatal check-ups at relevant obstetrics and gynecology services during 12, 24, and 32 weeks of pregnancy. Participants were examined a second time during their last routine prenatal hospital appointment and their resulting periodontal status was recorded. Changes in the number of teeth were also registered, alongside the presence of any caries. Women who had undergone dental treatment such as tartrectomy or periodontal treatment between the first and second examinations were excluded from analyses.

### Statistical analysis

The normality of continuous variables was assessed using the Kolmogorov–Smirnov test. Qualitative variables were described as absolute frequencies and percentages, whilst numerical variables were described as means and standard deviations or medians and interquartile ranges based on whether data met assumptions of normality. Bivariate analyses were performed to compare differences between participants’ sociodemographic characteristics and oral health status during the first and third trimesters of gestation. Qualitative variables were analyzed using the McNemar test while numerical variables were analyzed using the Student t-test for paired samples (paired t-test) or the Wilcoxon test depending on the normality of the variables. Obesity has been dichotomized in obese (BMI ≥ 30 kg/m^2^) vs. non-obese (BMI < 30 kg/m^2^).

Variables associated with a *p*-value < 0.10 following bivariate analysis were included in the multivariate analysis, in which oral health status during the third trimester of gestation was considered as the dependent variable. Variable selection was conducted using a backward stepwise selection process. Variables were retained if they were associated with a *p*-value > 0.10 at each step and the addition of each variable to the model was evaluated at each step to consider whether corresponding likelihood ratio test outcomes were significant. In the final statistical model, adjusted odds ratios and their 95% confidence intervals were calculated. All analyses were conducted using the software IBM SPSS Statistics v.19.

## Results

A total of 147 pregnant women completed the follow-up. Participant baseline characteristics (10–12 weeks pregnant) are presented in Table 1. The mean age of participants was 32.0 ± 4.4 years old. With regards to employment status, 98 (66.7%) women were in paid employment and 127 (86.3%) had an educational level corresponding to the completion of high school studies or higher. A total of 13 (8.8%) women were regular smokers, whilst 29 (19.7%) were obese based on BMI recorded at their first prenatal appointment.

**Table 1:** Baseline study sample descriptive characteristics (n = 147)

Variables	*n* (%)
Age (mean (SD))	32.05 (4.4)
Active workers	98 (66.7)
*Educational level*	
Elementary school	20 (13.6)
High school or above	127 (86.3)
Regular smokers	13 (8.8)
Body mass index at baseline (mean (SD))	25.5 (5.1)
*Obesity diagnostic*	
Underweight or normal weight	81 (55.1)
Overweight	37 (25.2)
Obesity	29 (19.7)
Diagnosed with diabetic or gestational diabetes	18 (12.2)

Data related to the progress of periodontal disease throughout pregnancy are presented in Table 2. Based on pre-specified definitions, periodontitis prevalence increased from 25 (17.0%) during the first trimester of gestation to 42 (28.6%) in the third trimester, whilst the observed prevalence of gingivitis increased from 54 (36.7%) to 63 (42.9%) between trimesters (*p* < 0.0001 in both cases). In addition, indices pertaining to periodontal clinical variables (CAL, BOP, and Plaque index) also significantly worsened between the two gestational periods.

**Table 2:** Evolution of periodontal disease between two gestational periods

Variables	First trimester *n* (%)	Third trimester *n* (%)	*p*-value
Periodontitis	25 (17.0)	42 (28.6)	*p* < 0.001^a^
Gingivitis	54 (36.7)	63 (42.9)	*p* < 0.001^a^
Plaque index^c^ (mean, SD)	0.95 (0.89)	1.99 (0.62)	*p* < 0.001^b^
CAL (mean, SD)	0.09 (0.41)	1.75 (1.31)	*p* < 0.001^a^
Pocket depth (mean, SD)	1.04 (1.11)	2.25 (0.97)	*p* < 0.001^a^

*CAL* Clinical attachment loss

^a^McNemar test for paired samples

^b^Student *t*-test for paired samples

^c^Used to assess oral hygiene

Associations between studied variables and the presence of periodontitis and gingivitis at both study periods are presented in Table 3. Obesity (32% vs 17.2%; *p* = 0.082), gestational diabetes mellitus (28% vs 9%; *p* = 0.016) and higher plaque indices (1.94 vs 0.75; *p* < 0.001) were more prevalent in the first trimester amongst women with periodontitis relative to healthy women. These variables were also associated with periodontitis during the third trimester of gestation. However, no significant differences in age, working status, or smoking habits were observed (Table 3). Working status and presence of bacteria plaques during the first trimester of gestation were the variables found to contribute most to gingivitis. In this sense, unemployed women had a lower prevalence of gingivitis compared to employed women (22.2% vs 39.8%; *p* = 0.022). Along the same line, women with worse plaque index in the first trimester of gestation suffered more frequently from gingivitis than women with better plaque index (1.39 vs 0.70; *p* < 0.001). With regards to the third trimester of gestation, gingivitis was more prevalent amongst women who regularly smoked than those who did not smoke (13.1% vs 3.3%; *p* = 0.032) (Table 3). Gingivitis and periodontitis were closely associated. In accordance with this, significant associations were found in relation to the prevalence of both conditions during the first and third trimesters of gestation.

**Table 3:** Frequency of periodontitis and gingivitis according to participant characteristics

*Periodontitis*						
Variable	First trimester			Third trimester		
	No periodontitis	Periodontitis	*p*-value	No periodontitis	Periodontitis	*p*-value
Age (mean; SD)	31.9 (4.4)	32.3 (4.6)	0.738	31.9 (4.3)	32.2 (4.7)	0.694
Unemployed (*n*, %)	41 (33.6)	8 (32.4)	0.538	36 (34.3)	13 (31.0)	0.427
Smoker (*n*, %)	10 (8.2)	3 (12.0)	0.385	7 (6.7)	6 (14.3)	0.127
Obesity^a^ (*n*, %)	21 (17.2)	8 (32.0)	0.082	15 (14.3)	14 (33.3)	0.010
Diabetes/ GD (*n*, %)	11 (9.0)	7 (28.0)	0.016	10 (9.5)	8 (19.0)	0.097
Plaque index^b^ 1T	0.75 (0.76)	1.94 (0.84)	*p*<0.001	0.63 (0.73)	1.76 (0-74)	*p*<0.001
Periodontitis 1T	–	–	–	3 (2.9)	22 (52.4)	*p*<0.001
Gingivitis 1T	54 (68.4)	0	*p*<0.001	34 (32.4)	20 (47.6)	0.062

*Gingivitis*						
	No gingivits	Gingivitis	*p* value	No gingivitis	Gingivitis	*p* value
Age (mean; SD)	31.6 (0.4)	32.7 (4.9)	0.332	31.8 (4.4)	32.2 (4.5)	0.576
Unemployed (*n*, %)	37 (39.8))	12 (22.2)	0.022	18 (28.6)	31 (36.9)	0.189
Smoker (*n*, %)	10 (10.8)	3 (5.6)	0.225	2 (3.2)	11 (13.1)	0.032
Obesity^a^ (*n*, %)	17 (18.3)	12 (22.2)	0.354	9 (14.3)	20 (23.8)	0.109
Diabetes/ GD (*n*, %)	14 (15.1)	4 (7.4)	0.134	5 (7.9)	13 (15.5)	0.129
Plaque index^b^ 1T	0.70 (0.91)	1.39 (0.65)	*p*<0.001	0.86 (0.81)	1.02 (0.94)	0.302
Periodontitis 1T	25 (26.9)	0	*p*<0.001	22 (26.2)	3 (4.8)	*p*<0.001
Gingivitis 1T	–	–	–	20 (23.8)	34 (54.0)	*p*<0.001

*GD* Gestational diabetes

*1T* First trimester

^a^Obese vs non-obese

^b^Used to assess oral hygiene

As shown in the multivariate analysis (Table 4), the most effective variable for predicting periodontitis in the third trimester of gestation was obesity (OR 2.834; 95% CI 0.919–8.74), oral hygiene (assessed in the first trimester of gestation using the plaque index) (OR 4.031; 95% CI 2.12–7.65) and the presence of periodontitis during early pregnancy (OR 15.104; 95% CI 3.60–63.36). On the other hand, the variable that most effectively predicted the presence of gingivitis in late pregnancy was suffering from gingivitis in the first trimester of gestation (OR 3.642; 95% CI 1.783–7.441). The overall accuracy of the final model was 91.2% and 65.1% for periodontitis and gingivitis, respectively.

**Table 4:** Multivariate analysis of periodontitis and gingivitis in the third trimester of pregnancy

Variables	Crude OR (95% CI)	*P* value	Adjusted OR (95% CI)	*p* value
Periodontitis obesity	3.0 (1.29–6.96)	0.011	2,83 (0.91–8.74)	0.070
Plaque index^a^ in first trimester	5.57 (3.14–9.87)	< 0.001	4.03 (2.12–7.65)	< 0.001
Periodontitis in first trimester	37.4 (10.21–136-95)	< 0.001	15.10 (3.60–63.36)	< 0.001
Gingivitis in first trimester	1.89 (0.91–3.94)	0.086		
Gestational diabetes	2.23 (0.81–6.13)	0.118		
Tabaco	0.42 (0.13–1.36)	0.151		
Age	1.01 (0.93–1.10)	0.681		
Unemployed	0.85 (0.39–1.85)	0.699		
*Gingivitis*				
Gingivitis in first trimester	3.75 (1.85–7.59)	< 0.001	3.642 (1.783–7.441)	< 0.001
Periodontitis in first trimester	0.14 (0.04–0.49)	0.002		
Obesity	0.53 (0.22–1.26)	0.533		
Tabaco	0.21 (0.04–1.02)	0.053		
Unemployed	1.46 (0.72–2.95)	0.290		
Gestational diabetes	0.47 (0.15–1.39)	0.175		

^a^Used to assess oral hygiene

## Discussion

In the present study, it was observed that the gingival and periodontal status of pregnant women aged 32 years on average deteriorated significantly between the first and third trimester of gestation. Oral hygiene in the first trimester assessed by plaque index was related to periodontitis in the third trimester but not with gingivitis, which proves the role of pregnancy in the development of non-dental plaque biofilm-induced gingival diseases. Moreover, in the multivariate model, obesity, poor oral hygiene, and periodontitis during the first trimester, explain the worsening of periodontitis that is observed towards the end of pregnancy. However, gingivitis is only explained by the gingival inflammation present since the beginning of the follow-up period.

There is extensive evidence that pregnancy and the corresponding increase in sex steroid hormone levels influence gingivitis [1–3, 9], and is not always directly associated with bacteria plaque [8]. Pregnancy and other conditions like genetic/developmental disorders, specific bacterial and viral infections, some inflammatory and immune conditions, etc., are included in the non-dental plaque biofilm-induced gingival diseases [8]. In accordance with this, the present research observed that the increased prevalence of gingivitis during the third trimester was not associated with the plaque index. Instead, it was related only to the presence of gingivitis during first trimester of gestation as indicated in the adjusted model. Although gingivitis in pregnant women is not always caused by dental plaque biofilm, the severity of the clinical manifestations often depends on plaque accumulation and subsequent gingival inflammation. For this reason, all healthcare providers must understand these interrelationships, inform patients of such conditions, and make appropriate referrals [5].

There is no consensus on the progression of periodontitis throughout pregnancy. As described in the literature, women presenting periodontitis before pregnancy have an approximately 15-fold increased risk of worsening their oral health status towards the end of gestation [22]. However, some authors claim that these findings of increased clinical attachment loss and probe depth (basic periodontal clinical variables), were the result of incorrect measurements of the inflamed gingiva caused by gingivitis, as well as reduced resistance to probe penetration [9]. In addition, the scientific community in the field of dentistry has tried to reach a consensus in terms of standardizing periodontitis assessments and case definitions [23] to reduce the high variability in prevalence results observed in different studies. In the present study, the percentage of women suffering periodontitis based on most recent diagnostic criteria [21] was higher during late pregnancy (28.6% in third trimester and 17% in first trimester). In any case, although the mechanisms responsible for the increased gingival inflammation and periodontitis observed during pregnancy are not fully understood, the mechanism underlying pregnancy-related deterioration in periodontal status seems to be microbiological. In this sense, pregnancy influences the composition of the oral microbiome, increasing periodontal pathogen counts (*Porphyromonas gingivalis* and *Aggregatibacter actinomycetemcomitans*, amongst others) and, in this way, promoting the onset of periodontitis [24]. Furthermore, it is clear that perturbations in neutrophil function, modifications in cellular and humoral immunity, hormone-induced changes in cellular physiology, and local effects on microbial ecology, altogether, play important roles in the overall process [7].

Analysis of the possible general and local conditions that influence periodontitis and gingivitis showed that obesity was one of the variables with the greatest impact on worsening the disease, but not the only one. Obese women had a 2.8-fold increased risk of developing periodontitis during late pregnancy compared to overweight and normal-weight women (95% CI = 0.91–8.74). In the present study, when adjusted by the rest of the variables, we observed that presenting periodontitis from the onset of pregnancy, having worse oral hygiene, and being obese, clearly explained the worsening of periodontal status at the end of pregnancy. Several studies have observed the impact of obesity on periodontitis, with this impact being attributed to several bioactive substances known as adipocytokines, which are secreted from adipose tissue and may directly injure periodontal tissue [25]. Also, it has been observed that this adipose tissue in overweight patients secretes typical proinflammatory mediators such as tumor necrosis factor-alpha, interleukin-6, and C-reactive protein [11]. The inflammatory mediators render the host more susceptible to inflammation in general [26] and, consequently, lead to the destruction of the periodontal tissues in face of the same amount of plaque accumulation [27]. Andrade et al. [28], observed that there is a significantly higher level of red complex pathogens, namely, *P.gingivalis* and *T.forsythia*, in the subgingival biofilm of women who are overweight or obese compared to normal weight. It would be of interest to determine whether the same occurs in young obese pregnant women.

It would seem reasonable believing that obesity and pregnancy may have a synergic effect on the appearance and progression of periodontal disease, although there is a scarcity of studies focused on this additive effect. Vogt et al. [29], observed that obese pregnant women had a 1.38-fold increased risk of developing periodontitis, whilst the observed OR was 4.06 in a study conducted by Lee et al. [30]. Differences between the present outcomes and those reported by these aforementioned studies could be again attributed to the definition of periodontal disease employed [30]. In our study, we have analyzed the presence of local and general conditions simultaneously and found in the multivariate analysis that oral hygiene (assessed by the observation of bacterial plaque), the presence of periodontitis, and obesity from the onset of pregnancy is associated with the worsening of periodontitis. For this reason, in our opinion, early odontological intervention is essential to prevent a worsening of periodontal status during pregnancy, especially in obese women.

Finally, the role played by gestational diabetes mellitus on the progress of periodontal disease throughout pregnancy was analyzed. Although the non-adjusted model showed that periodontitis diagnosed during the first visit to the hospital was associated with gestation diabetes mellitus (diagnosed during 24 weeks pregnant) in the study cohort, this association was non-significant in the multivariate model. The lack of statistical significance may be attributed to the close correlation that exists between obesity and gestational diabetes mellitus, in combination with the low number of pregnant women included in the study cohort with pre-gestational diabetes mellitus or gestational diabetes mellitus. While there is broad evidence regarding the bidirectional association between periodontal disease and diabetes in the general population and pregnant women [31, 32], the exact mechanism behind this association remains unclear [33]. The systemic proinflammatory status caused by periodontitis could be linked to physiopathological processes that are inherent to gestational diabetes mellitus [34]. Probably, the combined effect of systemic disorders, such as obesity and gestational diabetes mellitus, and the consequent systemic proinflammatory status, alongside increased oxidative stress is associated with the exacerbation of periodontal disease observed in some studies alongside increased oxidative stress, are associated with the exacerbation of periodontal disease observed in some studies [35]. In a recent population-based clinical study, the authors established a connection between periodontitis in pregnant women and gestational diabetes mellitus [13]. The results showed that the incidence of gestational diabetes mellitus in the patients with untreated periodontitis was significantly higher than in the non-periodontitis participants (11.21% vs. 4.79%) and that untreated periodontitis might be a potential risk factor for the occurrence of gestational diabetes mellitus (OR = 2.543, 95% CI = 1.612–4.012). These results, as it happens with ours although without being conclusive, highlight the importance of preventing periodontitis before and during pregnancy.

There is broad evidence regarding the lack of oral healthcare during pregnancy and its potential negative implications for both the mother and the newborn [36, 37]. Furthermore, it has been hypothesized that once the inflammatory cascade is activated during pregnancy, interventions targeting this pathway may be ineffective at reducing preterm birth rates [38]. For these reasons, women’s healthcare providers must engage their patients in oral health interventions and provide advice promoting the prevention of this pathology [39]. Institutions such as the American College of Obstetricians and Gynecologists recommend that all healthcare providers assess oral health at the first prenatal visit with the main purpose of checking periodontal and gingival conditions [5]. In addition to prenatal routine check-ups in southern Spain, where the present study was conducted, public oral health programs are available for pregnant women. These provide them with free dental cleaning and counselling regarding oral hygiene and non-cariogenic diets. However, although most health professionals are aware of the importance of oral health, they do not usually address it as part of prenatal care [40]. In addition, health science study plans in Spain usually lack theoretical and practical oral health competencies. Both academic and health institutions should ensure that oral health is present in the routine care of pregnant women with the aim of preventing the adverse pregnancy and maternal outcomes shown in the present research and other studies.

Several limitations must be acknowledged in relation to this study. Sample size limitations may have meant that some relevant results (i.e., associations with gestational diabetes mellitus) were not statistically significant. Participants lost to follow-up were correctly identified but their periodontal and gingival status during late pregnancy was unknown. This may have led to an underestimation of outcomes. Overlap in the pathophysiology of the different conditions analyzed (periodontitis, pregnancy, obesity, and gestational diabetes mellitus), along with their complex interactions, might limit the interpretation of present findings.

## Conclusions

Despite the limitations of the present study, findings support the hypothesis that pregnancy influences the exacerbation of periodontitis and gingivitis throughout this period, with obesity and oral hygiene during early pregnancy being the most important risk factors for the progression of these oral health conditions. It is important for non-dental professionals involved in the care of pregnant women to know and identify these risk groups to refer them to a dentist and/or implement early preventive measures to ensure adequate oral health in pregnant women.
